# Massive hemorrhage from the posterior intercostal artery following lower partial sternotomy

**DOI:** 10.1186/s13019-021-01718-1

**Published:** 2021-11-21

**Authors:** Masashi Hattori, Yu Matsumura, Fumitaka Yamaki

**Affiliations:** Department of Cardiovascular Surgery, Nagano Chuo Hospital, 1570 Nishi-tsurugamachi, Nagano, Nagano 3800814 Japan

**Keywords:** Lower partial median sternotomy, Post-sternotomy hemorrhage, Posterior intercostal artery bleeding, Sternal retraction, Bleeding

## Abstract

**Background:**

Median sternotomy remains the most common approach in cardiovascular surgery. Recently, minimally invasive procedures, such as minimally invasive cardiac surgery, robot surgery, and catheter therapy have been developed in cardiovascular surgery. However, all these surgeries cannot be performed by minimally invasive approaches. Several complications associated with median sternotomy have been reported, although post-sternotomy hemorrhage from the posterior intercostal artery is extremely rare.

**Case presentation:**

We present a case of posterior intercostal artery bleeding following lower partial sternotomy. A 79-year-old man underwent aortic valve replacement using lower partial median inverted L-shaped sternotomy that cut into the right second intercostal space. A postoperative chest radiograph indicated a hematoma in the right upper chest wall and pleural effusion. Hence, we inserted a drainage tube immediately. Approximately 2 hours after the surgery, his blood pressure gradually decreased. Blood drainage was observed from the tube, and the amount of blood drainage was not large. Contrast-enhanced computed tomography revealed a huge hematoma and hemorrhage from the fourth right posterior intercostal artery. Immediately, we performed emergency surgery. The lower partial sternotomy was repeated. We detected the origin of the bleeding that was identified in the right fourth posterior intercostal artery, and the bleeding was stopped. The postoperative course was uneventful.

**Conclusions:**

This case highlights the possibility of intraoperative bleeding from the intercostal artery, even in the absence of clearly rib fracture. In our case, we did not identify the cause of bleeding, although we suggest the inhomogeneous stress on the posterior ribs upon attaching the sternal retractor for lower partial sternotomy may have affected the posterior intercostal artery.

## Background

Median sternotomy remains the most common approach in cardiovascular surgery. Recently, minimally invasive procedures, such as minimally invasive cardiac surgery, robot surgery, and catheter therapy have been developed in cardiovascular surgery. However, all these surgeries cannot be performed by minimally invasive approaches. Several complications, such as infection, bleeding, pain, and fracture, were associated with median sternotomy in previous reports [1, 2]. Compared to the minimally invasive procedures, median sternotomy is inferior in terms of infection and cosmetic reasons, although it provides a wide and good surgical site, and remains a widely used method. Bleeding from veins, anterior intercostal arteries, and internal thoracic artery are often problems in post-median sternotomy bleeding. To the best of our knowledge, only a few cases of post-sternotomy hemorrhage from the posterior intercostal artery have been reported [3, 4]. Herein, we report a rare case of posterior intercostal artery bleeding following lower partial sternotomy.

## Case presentation

A 79-year-old man had a history of hypertension, chronic kidney disease, distal gastrectomy due to gastric cancer at 62 years of age, and right spontaneous pneumothorax at 63 years of age. Aortic regurgitation was identified from a heart murmur and was followed up by a nearby doctor. He had no shortness of breath; however, he had gradually decreasing cardiac function and exhibited significant depression of aortic valve regurgitation. He was referred to our hospital for cardiac surgery. Doppler echocardiography showed severe aortic regurgitation and the right coronary cusp prolapsed toward the left ventricular outlet trunk in diastole and aortic valvular prolapse. There was no cutaneous abnormality upon physical examination. No major abnormality was found in the preoperative laboratory results.

**Fig. 1:**
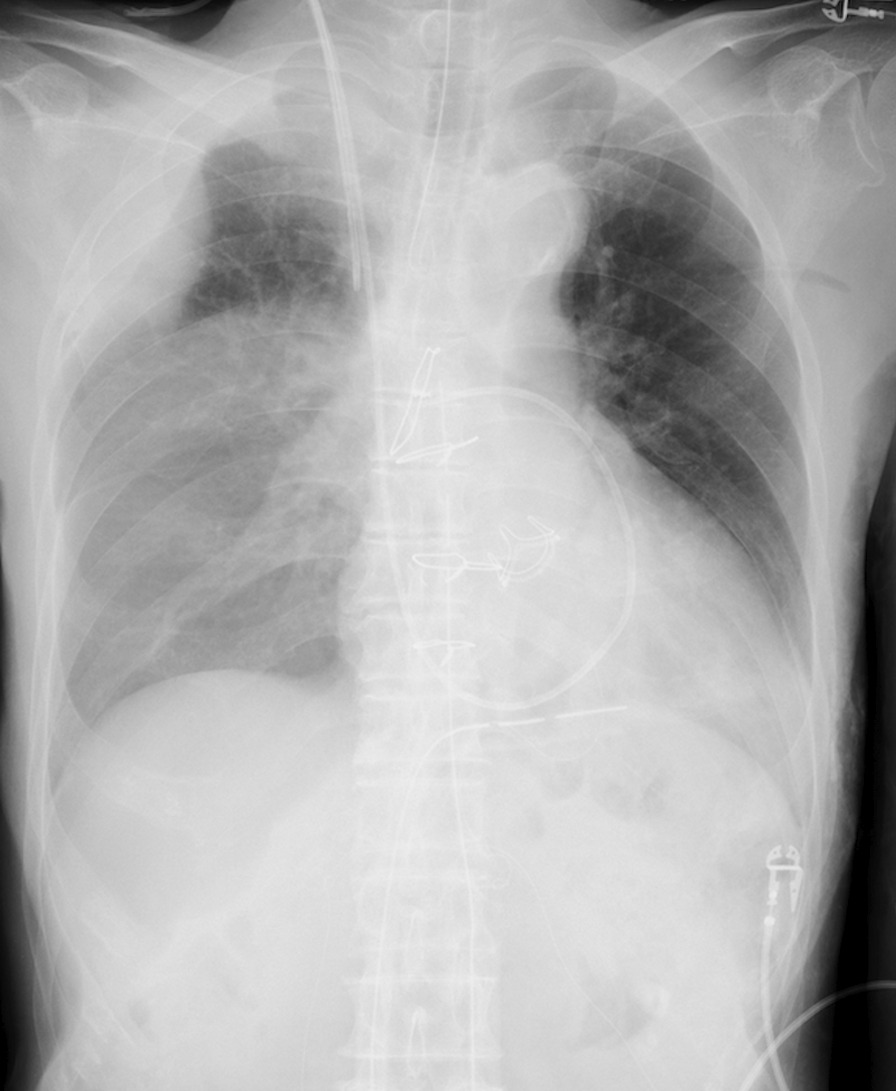
Postoperative chest radiograph showed a hematoma on the right upper chest wall and hemothorax

We performed cardiac surgery via a lower partial median inverted L-shaped sternotomy that cut into the right second intercostal space. Since the right internal thoracic artery was exposed and stretched, we ligated and transected it. Cardiopulmonary bypass was established with right atrial drainage and ascending aorta return. After cooling to 33℃, the ascending aorta was clamped under ventricular fibrillation, an aortotomy was made, and the selective coronary perfusion resulted in cardiac arrest without problems. We replaced the aortic valve with a 23-mm bioprosthetic valve. Weaning from the cardiopulmonary bypass was without difficulty. The surgery was completed without blood transfusion. The patient’s postoperative blood pressure and heart rhythm were stable. However, a postoperative chest radiograph indicated a hematoma in the right upper chest wall and pleural effusion (Fig. 1). This site did not appear to be damaged during the operation, although it was thought that the cause was blood dripping due to damage to the pleura during the operation. Therefore, we inserted a drainage tube immediately and transferred him to the intensive care unit.

**Fig. 2:**
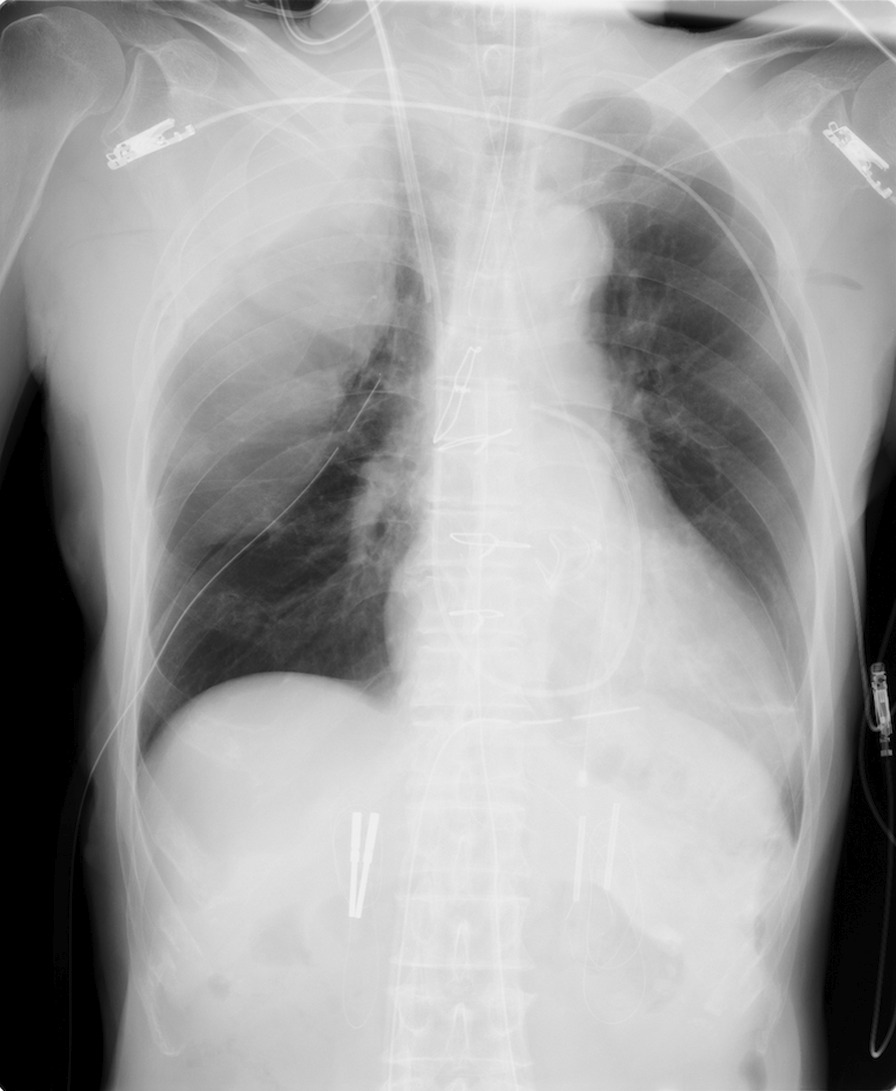
Enlargement of the hematoma before the re-operation

**Fig. 3:**
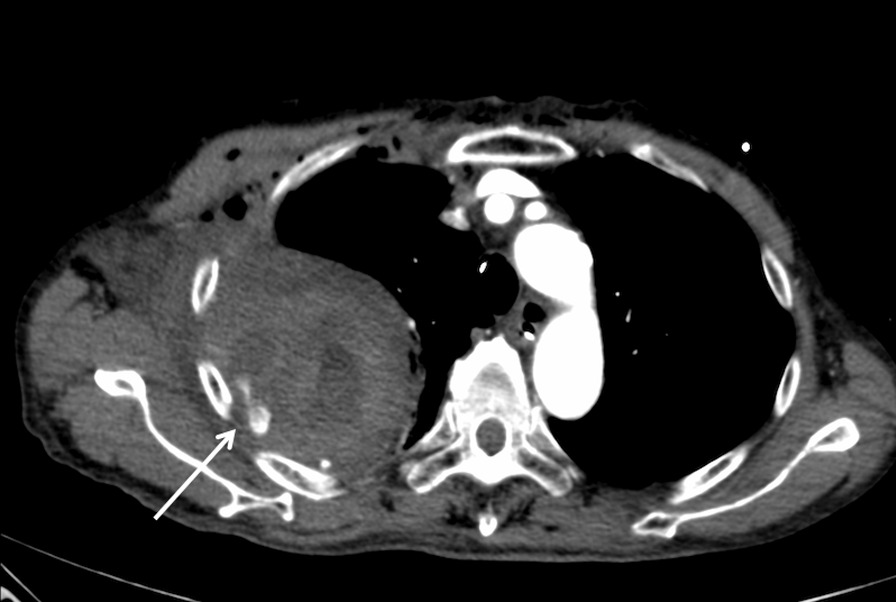
Contrast enhanced computed tomography showed hemothorax with bleeding from the right fourth posterior intercostal artery (arrow)

Approximately 2 hours after the surgery, the patient became anemic, and his blood pressure gradually decreased. This prompted us to transfuse blood. A small amount of blood drainage from the tube was observed. Blood retention was suspected and subsequently confirmed by Doppler ultrasounds. Then, chest radiography was performed again; findings were compared with the first postoperative chest radiograph. The second radiograph indicated enlargement of the hematoma (Fig. 2). Considering the possibility of iatrogenic bleeding, we removed the central venous catheter and the Swan-Ganz catheter immediately. We observed a gradual drop in his blood pressure; however, we performed contrast-enhanced computed tomography (CT). This revealed massive hemorrhage and extravasation from the fourth right posterior intercostal artery (Fig. 3), and no obvious fractures were found under bone conditions of the CT. Bleeding was immediately stopped because the patient was in shock. We performed emergency surgery through the previous lower partial sternotomy. We made an incision in the right visceral pleura, evacuated the 275-g hematoma from the cavity, and examined it carefully. The origin of the bleeding was identified in the fourth right posterior intercostal artery, although no rib fracture was noted on palpation of the lesion. The artery was cauterized with an electric scalpel and ligated. The patient’s postoperative course was uneventful, with no resumption of bleeding.

## Discussion and conclusions

Trauma including blunt chest and iatrogenic injuries is the most common cause of intercostal artery bleeding [5]. Spontaneous intercostal artery bleeding is generally observed in patients with disorders, such as neurofibromatosis type 1, systemic lupus erythematosus, or coarctation of aorta [6–8]. In our case, the CT did not reveal any rib fractures or evidence of trauma. Moreover, the patient did not have any remarkable medical history, and his physical examination and laboratory data did not suggest the presence of the above-mentioned diseases.

Median sternotomy has been the most common approach in cardiovascular surgery. Complications related to median sternotomy have been reported previously. However, intercostal artery bleeding following median sternotomy is extremely rare. To our knowledge, only two reports have described intercostal artery bleeding following median sternotomy. Alonso et al. reported the first case of intercostal artery bleeding [3]. In their case, the left second anterior intercostal artery developed a pseudo-aneurysm involving a sternal wire during sternotomy closure. Soquet et al. described two cases of intercostal artery bleeding [4]. The bleeding in the first case was from the left ninth posterior intercostal artery, and the other was from the right 12th posterior intercostal artery. The bleeding in these cases was spontaneous following cardiac surgery via a median sternotomy and was stopped by repeated sternotomy or transcatheter arterial embolization (TAE). In their cases, the authors could not identify the cause of the bleeding. In previous reports, most intercostal artery bleeding due to causes other than trauma was from the lower intercostal artery of the ninth or lower. Jang et al. showed the risk factors of intercostal artery bleeding, such as being elderly, female, and on anticoagulation medication, and having a cough [9]. In our case, only being elderly is applicable. Soquet et al. [4] suggested that the vessel wall weakness of the lower intercostal artery contributes to bleeding. However, in our case, bleeding was from the fourth intercostal artery and higher intercostal artery. Thus, it may be a different cause than those in the previous reports. Here, the bleeding site was not the surgical operation site, and the intraoperative hemodynamics were stable. Considering that postoperative hemodynamics became unstable, the bleeding may have occurred between the end of the cardiopulmonary bypass and the end of the surgery. This anatomically superior source of bleeding may be due to the incision associated with lower partial sternotomy. It is plausible that inhomogeneous stress on the posterior intercostal artery when we attached the sternal retractor for lower partial sternotomy or when we hooked the sternum wire for closing sternotomy affected the fourth posterior intercostal artery. Another cause of the bleeding may be the pleural adhesions that formed following spontaneous pneumothorax, which may have generated tension on the fourth posterior intercostal artery. If these are the causes, traumatic and iatrogenic bleeding is possible, although the cause remains unknown after all. Therefore, the possibility of bleeding should be considered when lower partial sternotomy is performed, and surgeons should be prepared to stop the bleeding, if necessary.

In our case, contrast-enhanced CT was used to accurately diagnose the bleeding source, which led to prompt treatment thereafter. Therefore, despite various diagnostic modalities, the CT is mandatory. For the treatment of intercostal artery bleeding, several reports have recently underscored the efficacy of TAE for hemostasis [10, 11]. Moreover, TAE is minimally invasive and is considered useful as it can be performed immediately, although the possibility of rebleeding remains. In fact, the case of Soquet et al. also had rebleeding after TAE, and there were cases where thoracotomy was performed for hemostasis [4]. Therefore, if the origin of bleeding can be diagnosed by CT, it may be useful to performed re-sternotomy for reliable hemostasis after cardiac surgery via median sternotomy. In our case, the patient was under shock and had undergone lower partial sternotomy. Because the bleeding could be identified easily via the re-sternotomy, we stopped the hemorrhage immediately.

In conclusion, post-sternotomy hemorrhage from the posterior intercostal artery is extremely rare. Although the cause of the bleeding was not identified in our case, the inhomogeneous stress on posterior ribs when we attached the sternal retractor for lower partial sternotomy may have affected the posterior intercostal artery.

## Data Availability

The datasets supporting the conclusions of this article are included within the article.

## Abbreviations

CT: Computed tomography
TAE: Transcatheter arterial embolization
